# Use of Antegrade Coronary Oxygen Persufflation as a Strategy for Donor Heart Preservation

**DOI:** 10.21470/1678-9741-2023-0469

**Published:** 2025-03-13

**Authors:** Maksim O. Zhulkov, Dmitry A. Sirota, Ilya S. Zykov, Olga V. Poveshchenko, Maria A. Surovtseva, Irina A. Kim, Andrey V. Protopopov, Azat K. Sabetov, Khava A. Agaeva, Alexandr G. Makaev, Aleksandr P. Nadeev, Vladislav E. Kliver, Evgeniy E. Kliver, Alexander M. Volkov, Natalya A. Karmadonova, Yaroslav M. Smirnov, Alexey D. Limanskiy, Aleksandra R. Tarkova, Aleksandr M. Chernyavskiy

**Affiliations:** 1 Adult Cardiac Surgery Department, Meshalkin National Medical Research Center, Novosibirsk, Russian Federation; 2 Department of Cardiovascular Surgery, Novosibirsk State Medical University, Novosibirsk, Russian Federation; 3 Cell Technology Laboratory, Research Institute of Clinical and Experimental Lymphology – Branch of the Institute of Cytology and Genetics, Siberian Branch of the Russian Academy of Sciences, Novosibirsk, Russian Federation

**Keywords:** Oxygen Consumption, Organ Preservation, Cardioplegic Solutions, Heart Transplantation, Feasibility Studies, Siblings, Tissue Donors, Heart Arrest, Cardiac Output, Reperfusion

## Abstract

**Objective:**

To assess the technical feasibility and functional, metabolic, and structural myocardial integrity of the donor heart after four hours of direct coronary oxygen persufflation (COP).

**Methods:**

This research was carried out on three-month-old minipig siblings weighing 23-36 kg. Cardiac arrest was achieved by administrating two liters of Bretschneider’s cardioplegic solution (histidine-tryptophan-ketoglutarate [HTK]) (Custodiol^®^, Germany) into the aortic root. Orthotopic heart transplantation was performed after three hours of cardiac arrest.

**Results:**

A statistically significant decrease in cardiac output was observed in both groups (from 3.36 ± 0.36 l/min and 3.72 ± 0.52 l/min in the HTK group and modified HTK + COP to 2.35 ± 0.52 l/min and 2.15 ± 0.34 l/min, respectively) (*Р*<0.05). Differences between both groups were insignificant (*P*>0.05). Cardiac output was 2.99 ± 0.45 l/min and 2.48 ± 0.58 l/min (*Р*>0.05) in both groups after 120 min of cardiac recovery. Lactate dehydrogenase, creatine phosphokinase-MB, and troponin I changes in coronary sinus blood were significantly higher in the early reperfusion period. Statistical insignificance was observed between both groups (*P*>0.05). Myocardial oxygen consumption was 8.2 [7.35; 9.35] ml-О_2_/min/100 g and 7.7 [6.75; 10.12] ml-О_2_/min/100g in both groups (*P*>0.05). Histological examinations demonstrate no significant myocardial ischemic injury in the persufflation group.

**Conclusion:**

The study demonstrated technical feasibility and safety of direct coronary persufflation for four hours during ex vivo donor heart conditioning. However, no significant advantages of direct COP were observed over the standard cold preservation protocol.

## INTRODUCTION

Donor remoteness is the limiting criteria and remains the primary issue in cases of heart deficiency. Research continues for new strategies to maintain the viability of individual organs ex vivo for a longer period. Crystalloid preservation of the donor heart by Bretschneider’s solution is the most commonly used method. However, the four-hour preservation compromises the organ itself, especially in the older age group^[1,2]^. The approach has the highest risk factor for primary graft dysfunction and recipient death^[3]^. The risk increases twice in a year after transplantation due to increasing ischemia time from three to six hours, compared to 50death risk decrease, if the ischemia time was < 1 hour^[4,5]^. Ischemia time of > 4 hours significantly increases the risk of primary graft dysfunction. This manifested itself in an 8death risk increase within 30 days after transplantation, as well as higher mortality rate in five and 15 years after the procedure^[6]^.

The appropriate preservation method includes three main components: hypothermia, composition of the storage solution, and oxygenation^[7]^. Two out of three are variable. First things first, oxygenation has several challenges. Solution modification, including macroergs and buffers, had insignificant influence on the removal of metabolic waste products, as had been proven earlier. On the contrary, oxygenation had a huge influence. Numerous variations of adjuvant cardioprotectors had no relevant success, including a wide range of pharmacological, metabolic, and physical assets^[8]^.

Hemoglobin is an oxygen-transport protein in physiological environments. Therefore, continuous hardware perfusion of the graft with donor blood or macroergs is the most physiological approach to oxygen carriage for cardiomyocytes. The TransMedics system (Massachusetts, United States of America) is the first available device for donor heart transplantation during normothermic perfusion. The perfusate is a proprietary solution that includes addition of insulin, antibiotics, methylprednisolone, sodium bicarbonate, multivitamins, and donor blood^[9]^. However, this preservation method is very expensive and requires constant monitoring, which complicates transportation process^[10,11,12]^. It is worth emphasizing that the search for simple and cost-effective long-term preservation method is a current issue in transplantology. Magnus R. made a surprising observation during the isolated heart perfusion of a cat in 1902^[13]^. The emptying of the reservoir and oxygen supply under pressure in the coronary arteries did not result in asystole; instead, the heart was rhythmically contracting within nine minutes. That case was the first mention of maintaining heart function by transmitting oxygen through coronary arteries. Despite subsequent achievements related to heart preservation through oxygen transmission in coronary arteries, the term “persufflation” (coronary oxygen persufflation [COP]) has officially replaced the term “gaseous oxygen perfusion”^[14]^. Liver and kidneys persufflation relevance replaced heart persufflation from 1960 to 1990^[15]^. However, the relevance of prolonged heart preservation method through COP increased in the early 2000s. Results of several studies have proven the physiological possibility and effectiveness of prolonged (approximately 14 hours) heart conditioning through persufflation, including short periods of warm ischemia (approximately 16 minutes)^[15,16,17,18]^.

Despite results showing high efficacy of COP as a method of prolonged conditioning, the approach and its safety are still criticized by physicians. The aim of this research is to adapt the technical aspects of COP to the current clinical protocol of orthotopic heart transplantation and to evaluate its efficacy in comparison with cold heart preservation.

## METHODS

### Experimental Animals’ Preparation – Anesthesia

This research was carried out on three-month-old minipigs. Preparation of animals as well as animal management and care followed routine protocols of the European Convention for the Protection of Vertebrate Animals used for Experimental and other Scientific Purposes (Strasbourg, 18.03.1986). The protocol for conducting the research was approved by the local bioethics committee of the Meshalkin National Medical Research Center (protocol № 1 12.10.2020).

Premedication was performed intramuscularly in the lateral part of the neck using atropine and Zoletil^®^ 100 on the day of surgery. The dose was individually selected according to body surface area. Once sedated, the surgical field and the area for catheterization of neck vessels were prepared. The animals were then taken to the operating theatre, placed in the supine position and intubated. The internal jugular vein and common carotid artery were cannulated for measurement of arterial blood pressure (ABP) and central venous pressure (CVP). General anesthesia was performed with sevoflurane and myorelaxation (pipecuronium bromide). The animals were connected to an automatic ventilator Fabius^®^ Plus (Dräger, Germany), inspiration positive pressure was 20-30 cm of water, and expiration was 5-8 cm of water, as well with a tidal volume of 8 ml/kg and a frequency of 12-14 breaths per minute.

### Donor: Heart Extraction and Preservation Method

Minipigs weighing 33 ± 3.2 kg were premedicated as described above. The hearts were exposed by median sternotomy in all cases. A cardioplegic 7 Fr cannula was placed in the aortic root after administration of heparin at a dose of 3 mg/kg. The ascending aorta was cross-clamped after occlusion of the both caval veins and the left azygos vein in the control group (histidine-tryptophan-ketoglutarate [HTK], n=8), cold cardioplegia was administered through the aortic root with two liters of Bretschneider’s solution (HTK) (Custodiol^®^, Germany) at a pressure of 75 mmHg for the first minute and then at 40 mmHg for nine minutes. The hearts were then stored in an appropriate solution at a temperature of 0 to 1°C. In the experimental group (modified HTK [mHTK] + COP, n=8), the hearts also received continuous antegrade COP as described by Fischer J.^[19]^. Cardioplegia was administered with mHTK (addition of 40 mg/l of hyaluronidase). A silicon aortic valve guard was then placed, which was cut from glove rubber in the shape of a trefoil and fixed with a knot suture to prevent gaseous oxygen leakage. Carbogen (95O_2_, 5СО_2_) was then persufflated either through the ascending aorta or the brachiocephalic trunk, at an aortic root pressure of 40-45 mmHg. The hearts were immersed in a plastic bag filled with mHTK solution, which was placed in a container with ice. Drainage tubes were placed in the right and left ventricular cavities. The ends of these cannulas were left in the solution to determine gas leakage. After three hours of preservation, we started to prepare for transplantation.

### Recipient: Donor’s Heart Implantation

The hearts of minipigs weighing 25.17 kg were exposed by median sternotomy. After administration of heparin at a dose of 3 mg/ kg, the right common carotid artery and both caval veins were cannulated. Cardiopulmonary bypass (CPB) was then initiated. The donor heart was extracted with a wide cuff of pulmonary veins. The pigs were cooled to 28°C. Orthotopic transplantation of the donor heart was performed using bicaval technique: left atrium, pulmonary artery, aorta, and both caval veins were subsequently anastomosed. For immunosuppression, all recipients received pulse therapy with methylprednisolone (Methylpred^®^ Orion, Portugal) at a dose of 1500 mg before removing the aortic clamp. In the persufflation group, the implantation of the donor heart was performed with continuous antegrade COP up to the aortic end-to-end anastomosis. Heart reperfusion started with a 10-minute warm reflush (37°C) with modified Krebs-Henseleit solution at a pressure of 50 mmHg, containing 50 µmol/l of calcium and 15 µmol/l of adenosine to remove residual air from the coronaries. Samples from the arterial cannula and coronary sinus were analyzed for myocardial oxygen consumption and myocardial ischemia level markers during the first minutes of reperfusion. When 30 minutes passed, a myocardial biopsy of the apex was performed. Warming was performed gradually. The pigs were then weaned off CPB. We euthanized the pigs by administrating 100 ml of 4potassium chloride solution in a surgical level of anesthesia (propofol [4-7 mg/ kg], fentanyl [0.006-0.008 mg/kg], and inhalation of sevoflurane [2-4%]).

### Measurements

**Fig. 1:**
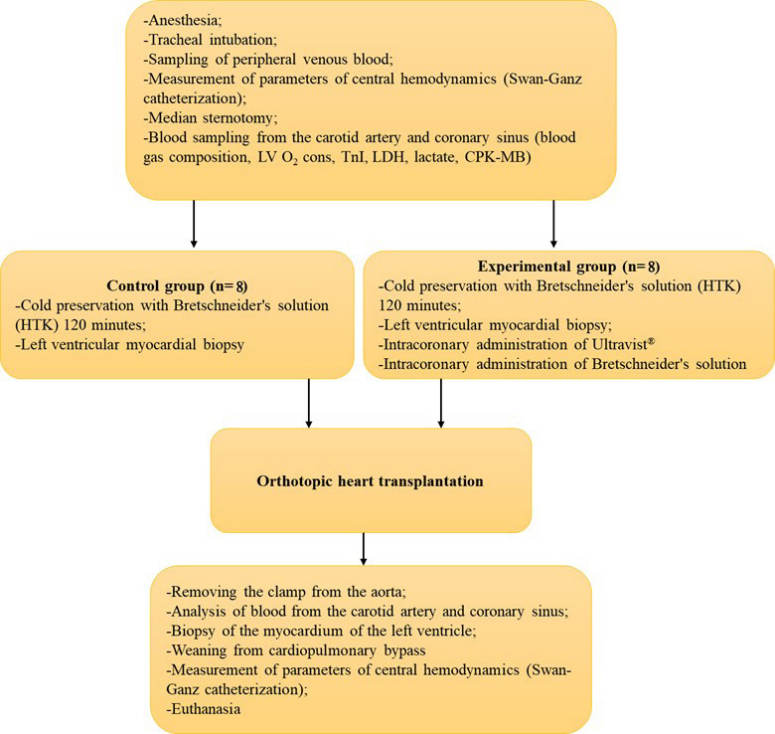
Experimental protocol. CPK-MB=creatine phosphokinase-MB; HTK=histidine-tryptophan-ketoglutarate; LDH=lactate dehydrogenase; LV O_2_ cons.=left ventricular oxygen consumption; TnI= troponin I.

During the experiment, we controlled ABP by cannulation of the left common carotid artery, CVP by cannulation of the right external jugular vein, heart rhythm disturbances (electrocardiogram), body temperature, blood gas composition, and activated clotting time. Epicystostomy was performed to control diuresis. Blood analysis was performed with a hematology analyzer Sysmex XT 4000i (Sysmex, Germany) according to the recommendations. Central hemodynamics were obtained by Swan-Ganz catheterization of the right heart. Initial measurements were taken immediately after the start of endotracheal ventilation. Final measurements were taken two hours after weaning off CPB as recommended by the protocol (Figure 1).

Physiological parameters were obtained with the IntelliVue MP70 system (Philips, Netherlands). Blood samples were taken from the coronary sinus to evaluate myocardial ischemia markers – troponin I (TnI), creatine phosphokinase-MB (CPK-MB), lactate dehydrogenase (LDH), lactate, and apex biopsy before and after the ischemia period according to the protocol. Myocardial oxygen consumption was calculated according to Formula 1 (Figure 2). Blood oxygen level was calculated in accordance with Formula 2 (Figure 3).

$$\text{LV}{\text{O}}_{2}\text{cons}.=\frac{\left({\left[{\text{O}}_{2}\right]}_{\text{a}}\right)-\left({\left[{\text{O}}_{2}\right]}_{\text{cs}}\right)\times \text{CAF}}{\text{LV mass}},{\text{ml-O}}_{2}/\min/100\text{g}.$$

**Fig. 2** – *Formula 1. CAF=coronary arteries flow; [O_2_]_a_ =arterial blood oxygen level; [O_2_]cs=coronary sinus oxygen level; LV=left ventricular; LV O_2_cons.=left ventricular oxygen consumption.*

$${\text{O}}_{2}=\frac{\%{\text{O}}_{2}\text{Sat}\times \left[\text{Hb}\right]\times {\text{O}}_{2}\text{capacity of Hb}\left(1.34\text{ml}-{\text{O}}_{2}/\text{g}\right)}{100},{\text{ml-O}}_{2}/\text{dl}.$$

**Fig. 3** – *Formula 2. Hb=hemoglobin.*

Myocardial samples were obtained from the apex for histological examination by placing them in 10 formalin on phosphate buffer (pH 7.4), and then samples were embedded in paraffin. Sections with a microtome (5 µm) (Microm™ HM 550) were obtained from the heart with hematoxylin and eosin staining according to van Gieson method, combining orcein for elastic fibers and periodic acid-Schiff stain reaction. General histological and morphometric data were obtained with the micro-complex software, light microscope (Carl Zeiss), an AxioCam MRc digital video camera, and Pentium^®^ 4 computer.

### Statistical Analysis

Statistica 10.0 software (StatSoft Inc., United States of America) was used for statistical analysis of the research. As descriptive statistics, mean ± standard deviation was presented for numerical variables. Normally distributed data were tested with Shapiro-Wilk. Further assessment was validated by Levene’s test. *t*-test was used for normally distributed data of numerical variables. Non-parametric methods were used in cases of non-normally distributed data. Statistical significance between the groups was established at *P*<0.05.

## RESULTS

**Fig. 4:**
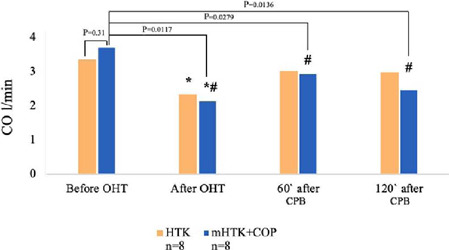
Cardiac output (CO). Function of four-hour preserved pig hearts before and after orthotopic heart transplantation (OHT). *Р<0.05 vs. before heart transplantation. #Р>0.05 vs. histidine-tryptophan-ketoglutarate (HTK)-group. COP=coronary oxygen persufflation; CPB=cardiopulmonary bypass; mHTK=modified histidine-tryptophan-ketoglutarate.

A total of 16 orthotopic transplantations were performed. Donor heart ischemia time was 248 ± 12 to 242 ± 10 minutes (*Р*>0.05) in the experimental and control groups, respectively. The average time of the procedure was comparable between groups: 47 ± 6 and 39 ± 7 minutes (*Р*>0.05). The reperfusion time was 60 ± 8 minutes in all cases. Cardiotonic infusion was then started (dopamine 10 mcg/kg/min, adrenaline 0.1 mcg/kg/min) while pigs were gradually weaned off CPB in all cases. Changes in cardiac output (CO) were assessed at three different time points: 1) right after weaning off CPB; 2) 60 minutes after weaning off CPB; and 3) 120 minutes after weaning off CPB. A statistically significant decrease in CO was observed in both groups compared to the baseline values (Figure 4) from 3.36 ± 0.36 l/min and 3.72 ± 0.52 l/min in the HTK group and mHTK + COP group to 2.35 ± 0.52 l/min and 2.15 ± 0.34 l/min (*Р*<0.05), respectively. However, differences between both groups were insignificant (*P*>0.05).

In the persufflation group, recovery of the cardiac pump function required active cardiotonic and antiarrhythmic support. Stable ventricular fibrillation was observed in all cases of mHTK + СОР, but the restoration of the correct sinus rhythm required several (about 10) defibrillation attempts. On the contrary, animals in the HTK group showed spontaneous restoration of sinus rhythm in all cases (Table 1).

**Table 1:** Results of myocardial extracts from left ventricle’s myocardium.

Group	Baseline	After OHT	*P*-value
HTK n=8	9.15 [7.17; 11.9]	8.2 [7.35; 9.35]	0.31
mHTK + COP n=8	10.6 [8.18; 15.42]	7.7^#^ [6.75; 10.12]	0.011

^#^*Р*>0.05 *vs.* HTK-group

Median and (Q1-Q3) matches values in brackets

COP=coronary oxygen persufflation; HTK=histidine-tryptophan-ketoglutarate; mHTK=modified histidine-tryptophan-ketoglutarate; OHT=orthotopic heart transplantation

**Fig. 5:**
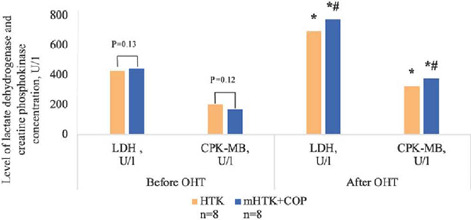
Level of lactate dehydrogenase (LDH) and creatine phosphokinase-MB(CPK-MB)concentrationbeforeandafterorthotopic heart transplantation (OHT). *Р<0.05 vs. before heart transplantation. #Р>0.05 vs. HTK-group. COP=coronary oxygen persufflation; HTK=histidine-tryptophan-ketoglutarate; mHTK=modified histidine-tryptophan-ketoglutarate.

**Fig. 6:**
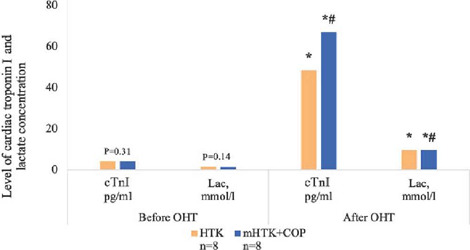
Level of cardiac troponin I (cTnI) and lactate concentration (Lac) before and after orthotopic heart transplantation (OHT). *Р<0.05 vs. before heart transplantation. #Р>0.05 vs. HTK-group. COP=coronary oxygen persufflation; HTK=histidine-tryptophan-ketoglutarate; mHTK=modified histidine-tryptophan-ketoglutarate.

Changes in LDH, CPK-MB, and TnI in coronary sinus blood are shown in Figures 5 and 6. Despite observed statistically significant myocardial ischemia markers level increase after the ischemia period, there was no statistically significant difference between the groups (*P*>0.05).

In the persufflation group, myocardial oxygen consumption was statistically significantly lower after reperfusion (*P*=0.011). However, there was not found significant difference in oxygen consumption between groups (*P*>0.05) (Table 2).

**Table 2:** Myocardial oxygen consumption (ml-О_2_/min/100g).

Measure/group	Control (n=8)		Experimental (n=8)	
	Before OHT	After OHT	Before OHT	After OHT
ATP, ng/ml (100 g/l protein conversion)	12.9 ± 4.2	13.1 ± 1.9	11.9 ± 1.9	13.2 ± 3.2
VEGF, pg/ml (100 g/l protein conversion)	7.9 ± 4.2	97.8 ± 2.7*	9.3 ± 2.3	10.1 ± 2.3^#^
NO, µM/mL (100 g/l protein conversion)	52.4 ± 23.6	37.8 ± 19.8*	53.6 ± 6.9	55.3 ± 5.9^#^
CK	38.9 ± 7.9	27.3 ± 5.5	36.2 ± 4.1	35.3 ± 8.6

*Р<0.05 *vs.* concentration before OHT; ^#^*P*>0.05 *vs.* control group

АТP=adenosine triphosphate; CK=creatine kinase; NO=nitric oxide; OHT=orthotopic heart transplantation; VEGF=vascular endothelial growth factor

The persufflation group showed no statistically significant changes in adenosine triphosphate, vascular endothelial growth factor (VEGF), nitric oxide (NO), and creatine kinase. In contrast to the control group, which showed a statistically significant increase in VEGF and NO decrease (Table 2).

**Fig. 7:**
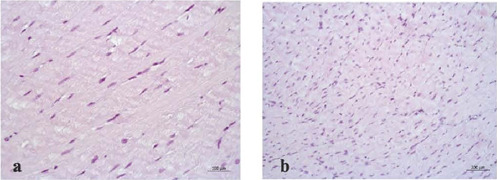
Light optical image of the myocardium of the left ventricle with the preservation of muscle fiber diameters and mild contractures. a) Histidine-tryptophan-ketoglutarate group, hematoxylin-eosin staining, magnification × 400; b) modified histidine-tryptophan-ketoglutarate + coronary oxygen persufflation group, hematoxylineosin staining, magnification × 200.

Histological findings of myocardial parenchyma and stroma in experimental and control groups were generally similar. Muscle fibers of normal size and muscle sarcoplasm were uniformly stained with hematoxylin and eosin (Figure 7). Transverse striations were clearly observed in the longitudinally sectioned fibers, as were areas of mild myofibril contracture. The nuclei of the muscle fibers were mostly medium-sized, oval, rod-shaped, or elongated. All the nuclei were stained dark blue with clumped chromatin.

**Fig. 8:**
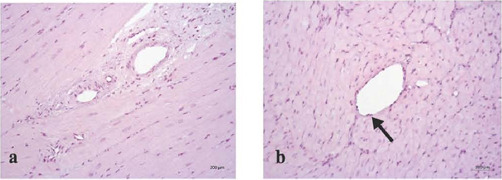
Light optical image of the intramyocardial vessels of the left ventricle, hematoxylin-eosin staining, magnification × 200. a) Histidine-tryptophan-ketoglutarate group, b) modified histidine-tryptophan-ketoglutarate + coronary oxygen persufflation group, preservation of the endothelial layer (shown by the arrow), hematoxylin-eosin staining, magnification × 400.

In the control group, the epicardial stroma was moderately and patchily edematous. Arteries and veins had wide oval lumen, perivascular edema, and isolated lymphocytes in capillaries. On the contrary, in the experimental group, the marginal pool of leukocytes was diffuse in nature. Individual cardiomyocytes had insignificant perinuclear edema, dilated vessels with persistent oval outlines. In both groups, endothelial cells were evenly divided, flat, and maintained their integrity (Figure 8).

## DISCUSSION

The four-hour ischemia time is the main factor limiting the number of potential donors and their capacity pool. Nowadays, the ischemia time for cold preservation is limited from three to five hours^[19]^. Unfortunately, modern cold preservation method requires that all the cell needs are met except for oxygen. Perfusion systems and hyperbaric oxygenation devices are not widely used by physicians due to their unwieldiness and high cost of consumables. The COP method, which was discovered more than a century ago, did not require complex perfusion equipment, unlike continuous oxygenated preservative perfusion solution or blood. Persufflation is a combined method involving primary cold cardioplegic preservation followed by continuous antegrade delivery of gaseous oxygen into the coronary arteries.

Despite the numerous studies demonstrating high efficacy of COP as a method of long-term (14 hours) graft conditioning, the method has not been widely adopted by clinicians^[20,21]^. Sabiston D. et al.^[22]^ performed the first studies showing the efficacy and safety of COP, and the first results demonstrating safety and efficacy were published in 1959. Sabiston D. et al.^[22]^ performed a series of experiments investigating antegrade COP (A-COP) using carbogen (95O_2_, 5СО_2_) and showed that dogs hearts were able to continue contracting for five hours (2.5-8 hours) ex vivo in normothermic conditions. The next stage of the experiment was A-COP in situ for 25-30 minutes, which eventually resulted in normal coronary blood flow. Hemodynamic function was restored in most cases. The main findings of the study showed that a heart can be supplied with gaseous oxygen by direct persufflation. Blood can be used to restore contractile function of the heart after A-COP and coronary reperfusion^[23]^.

Talbert J. et al.^[24]^ introduced the concept of retrograde COP (R-COP) in 1960. Retrograde perfusion of the oxygenated blood through the coronary sinus was widespread method to support heart rhythm and protect against short-term ischemia time during open interventions on the aortic valve surgery^[25]^. The authors injected carbogen through the coronary sinus which resulted in heart rhythm support for an average 3.5 hours, or up to 5.5 hours in cases that additional cannulation of the anterior cardiac veins was required. Camishion R et al.^[25]^ published an article in 1966 about their experience with R-COP practice during aortic valve interventions^[25]^. The term “persufflation” was officially replaced by the term “gaseous oxygen perfusion” in 1971^[14]^, resulting in a significant decrease in the interest in persufflation^[15]^.

Persufflation became again a subject of research in the 1990s. Kuhn-Reigner F. et al.^[18]^ published the research on the use of A-COP as a method of conditioning of the heart before orthotopic allotransplantation during the experiment in 1998^[18]^. Fischer J et al.^[17]^ carried out a similar experiment. The average ischemic time in these experiments was 14.5 hours. The authors described significant benefits in CO, coronary blood flow, left ventricular pressure, and recovery of myocardial relaxation after long period of A-COP compared to the isolated cold preservation group^[21]^. During the experiment, no negative influence of COP on the recovery of pump function was observed compared to the control group. Myocardial ischemia markers in coronary blood flow such as LDH, TnI, CPK-MB, and lactate were reliably above baseline in both persufflation and control groups. Such results were associated with anatomical features of the animals’ hearts such as hypertrophy, myocardial stiffness, diastolic dysfunction, and reduced left ventricular cavity, all of which could have caused inadequate myocardial protection by Bretschneider’s solution (Custodiol^®^, Germany) and a reduction of myocardial contractility after reperfusion. In addition, the possible influence on the results of the lack of donor-recipient cross-matching resulting in graft-versus-host disease was not considered. Histopathological examination also showed no significant myocardial ischemic injury with persufflation compared to the control group. The endothelial cells maintained their integrity and patency.

In view of these results, it remains unclear why COP is not widespread. Probably “the barrier” of direct gas injection into the coronary arteries remains a source of skepticism about the safety of COP, based on the risk of embolism. This opinion is widespread among physicians. Persufflation has no effect on transplantation and does not complicate it in view of the continuous gas supply to the aortic root.

### Limitations

This study has several limitations. Firstly, there were a small number of animals. However, this did not prevent the achievement of statistically significant results, emphasizing the disparity that existed. Although the minipigs were matched for swine leukocyte antigen class I antigen and taken from the same litter, some degree of biological variation among animals was inevitably present and may have influenced the final result. In this experimental model of heart transplantation, Bretschneider’s solution (Custodiol^®^, Germany), was used as a preservation solution, but the individual sensitivity of the preservative solution on the hearts and the technique were not studied.

## CONCLUSION

During the conditioning phase of the experiment, the technical feasibility and safety of direct COP in ex vivo donor heart was demonstrated. However, the experiments showed no advantage of the method four hours after the onset of donor heart ischemia compared to the standard cold preservation protocol using Bretschneider’s solution. The similarity in functional, biochemical status, and graft integrity between groups could also be the result of the short ischemia period and observation as well, which requires long-term research.
